# Malignant melanoma without primary, presenting as solitary pulmonary nodule: a case report

**DOI:** 10.1186/s13256-021-02933-z

**Published:** 2021-07-16

**Authors:** George Tsaknis, Muhammad Naeem, Advitya Singh, Siddharth Vijayakumar

**Affiliations:** 1 Department of Respiratory Medicine, Lung Cancer Service, Kettering General Hospital NHS Foundation Trust, Kettering, UK; 2 Department of Radiology, Kettering General Hospital NHS Foundation Trust, Kettering, UK

**Keywords:** Malignant melanoma, Solitary pulmonary nodule, Needle biopsy, Intramuscular metastasis, Case report

## Abstract

**Background:**

Solitary pulmonary nodules are the most common incidental finding on chest imaging. Their management is very well defined by several guidelines, with risk calculators for lung cancer being the gold standard. Solitary intramuscular metastasis combined with a solitary pulmonary nodule from malignant melanoma without a primary site is rare.

**Case presentation:**

A 57-year-old white male was referred to our lung cancer service with solitary pulmonary nodule. After positron-emission tomography, we performed an ultrasound-guided core needle biopsy of an intramuscular solitary lesion, not identified on computed tomography scan, and diagnosed metastatic malignant melanoma. The solitary pulmonary nodule was resected and also confirmed metastatic melanoma. There was no primary skin lesion. The patient received oral targeted therapy and is disease-free 5 years later.

**Conclusions:**

Clinicians dealing with solitary pulmonary nodules must remain vigilant for other extrathoracic malignancies even in the absence of obvious past history. Lung metastasectomy may have a role in metastatic malignant melanoma with unknown primary.

## Background

Solitary pulmonary nodules (SPN) are the most common finding in chest imaging, and their detection rate is increasing logarithmically as populations start attending computed tomography (CT) lung cancer screening programs worldwide. In a population that is “high risk” for developing lung cancer, nodules are detected in almost 50% of individuals, depending on agreed local “cut-off” points for their reporting. The majority of these nodules are small and benign, but some will still be malignant. Early treatment has been clearly associated with lung-cancer-specific mortality reduction of up to 20% and all-cause mortality reduction of 6.7% [1]. However, the possibility of solitary metastases from an extrathoracic cancer should not be overlooked, as these can be confirmed in as high as 10% of the cases [2, 3, 4]. According to the latest British Thoracic Society guidelines, in high-risk patients with high probability of malignancy, SPNs should be investigated and treated rapidly [5]. The cornerstone investigation is positron-emission tomography CT (PET CT) to guide radical treatment in individuals with good performance status.

Malignant melanoma (MM) accounts for less than 2% of all skin cancer cases, but is still accountable for approximately 75% of all skin cancer mortality [6]. It is highly curable if detected early, with an overall 5-year survival of 98% (localized), and has a predilection for metastasis early, with lung and pleura being the most common sites [7]. Metastatic melanoma without a primary lesion is even more uncommon (~2–9% of all MM cases), and complete regression of the primary tumor is one of the proposed mechanisms of such presentations, mainly in male patients [8].

## Case presentation

**Fig. 1:**
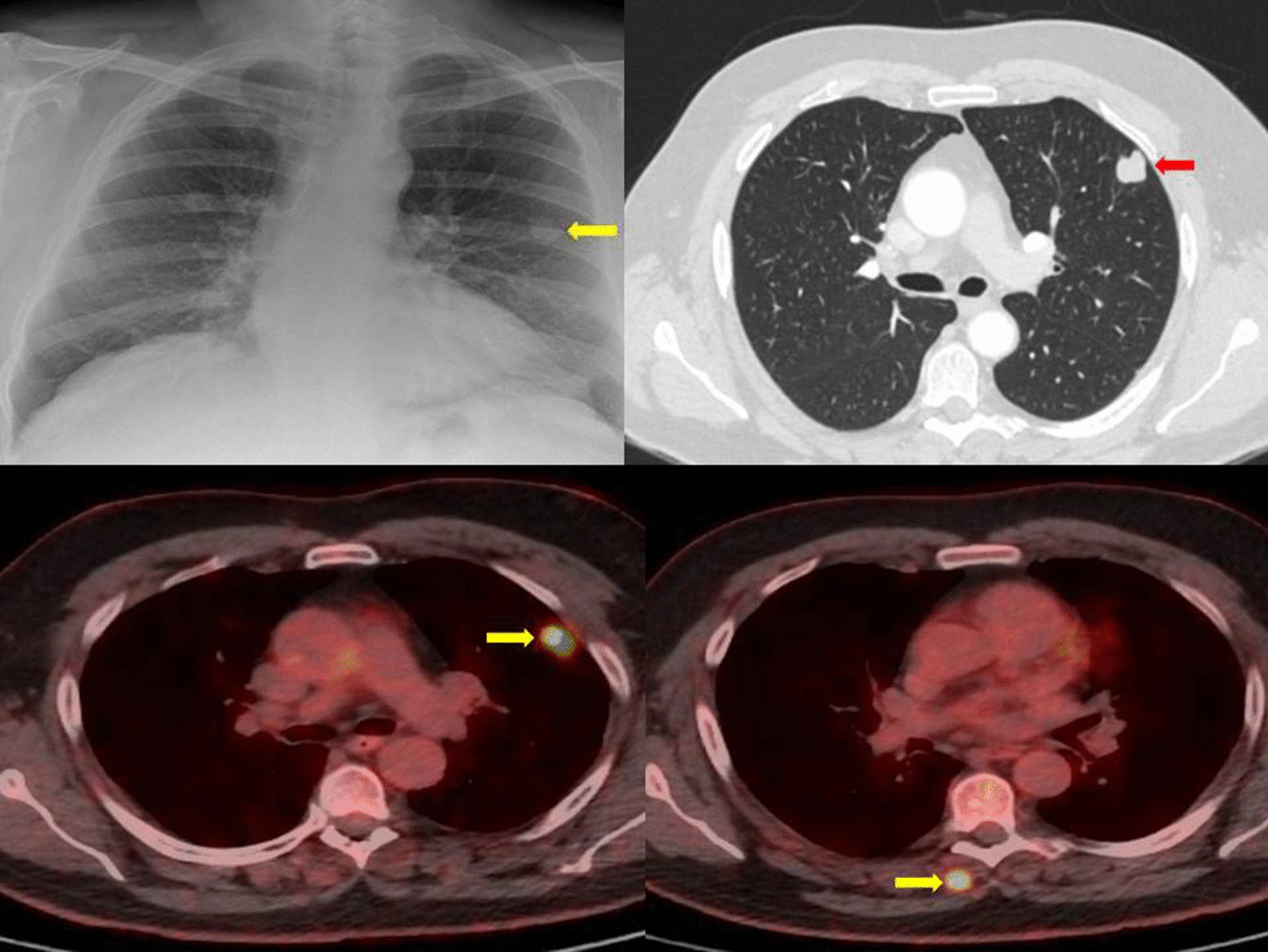
Chest X-ray showing a “coin lesion” in the left mid-zone (yellow arrow). CT section confirming the left-upper-lobe 20 mm solid (red arrow), non-spiculated, solitary nodule. Sections of PET showing high SUV activity of the lung lesion and a separate solitary posterior right paravertebral intramuscular lesion (erector spinae) at the level of T6 vertebral body, not visible on CT (yellow arrows)

A 57-year-old white male, current heavy smoker, was referred urgently to our lung cancer service as “suspected lung cancer” following an abnormal chest X-ray performed to investigate a cough. The X-ray revealed a “coin lesion” in the left middle zone (Fig. 1), and there was no previous imaging to compare. The patient had a strong family history of lung cancer (father and brother), and a personal medical history of hypertension (on amlodipine). He denied any pains, there were no palpable lymph nodes, no skin lesions, chest auscultation was clear, and there was no finger clubbing. Given the strong family history of lung cancer with excessive smoking, the primary differential diagnosis of lung cancer was considered. It was approached as a straightforward case of SPN, and we urgently organized a CT staging scan (Fig. 1), which reported a left-upper-lobe 20 mm solid, non-spiculated, solitary nodule, without emphysematous changes, no enlarged lymph nodes, and no other extrathoracic metastases. The provisional staging for lung cancer was T1b N0 M0 (BROCK risk 28.1%). His PET CT a few days later confirmed a PET-avid 20 mm left-upper-lobe lesion (SUVmax 9.6), but surprisingly also a solitary posterior right paravertebral intramuscular lesion (erector spinae) at the level of T6 vertebral body, with SUVmax 10.1, which was not clearly visible on the staging CT scan, even with the benefit of hindsight (Fig. 1). There were no enlarged or PET-avid mediastinal or hilar lymph nodes.

**Fig. 2:**
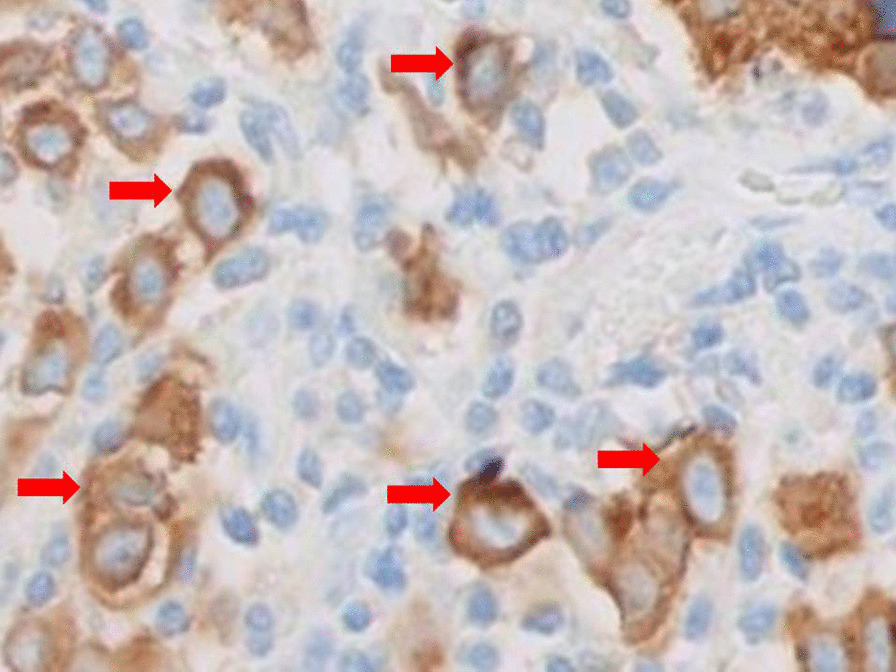
Cytoplasmic positivity for Melan-A in tumor cells (red arrows). Stain on the needle core biopsy histological sample from the posterior right paravertebral intramuscular lesion

In our lung cancer service, we run a physician-led rapid access ultrasound (US)-guided biopsy service, and with the patient’s consent, we proceeded with a “same day” ultrasound-guided needle core biopsy of the paravertebral intramuscular lesion. Four cores of pale tissue were obtained under guidance, with 18G Tru-Cut needle, without complications. Histopathology revealed presence of tumor with associated fibroadipose tissue and skeletal muscle, with some of the malignant cells showing a plasmacytoid morphology. There was widespread nuclear atypia with hyperchromasia and pleomorphism and evident mitotic activity. Immunohistochemistry showed positive S100, Melan-A (Fig. 2), and patchy pattern of human melanoma black-45 (HMB-45). Tissue was negative for AE1/AE3, CK 8/18, CK 5/6, CD45, CD138, napsin A, and thyroid transcription factor 1(TTF-1). The p-63 was positive, but the more specific marker of squamous cell cancers (SCC) p-40 was negative, steering the diagnosis further away from SCC. The combination of the morphology and immunoprofiling was consistent with a diagnosis of metastatic malignant melanoma (T0 N0 M1). His serum lactate dehydrogenase (LDH) levels were normal. BRAF testing was immediately performed, and detected a “c.1799T>A p.(Val600Glu)” mutation, with ~90% tumor content. Following discussions between the melanoma and the lung cancer multidisciplinary team meetings, and normal investigations from ophthalmology, ear, nose, and throat (ENT), upper and lower gastrointestinal endoscopy, bronchoscopy, magnetic resonance imaging (MRI) brain, and ultraviolet (UV) lamp examination, we decided to separately resect the SPN, in view of the high risk of a primary lung cancer and the absence of a primary melanoma. The patient had a video-assisted thoracoscopic (VATS) wedge resection of the peripheral 20 mm lesion, with clear margins of 9 mm (V0 R0) and had the same histopathological findings and immunoprofile, confirming MM. The patient did not consent to additional excision of the intramuscular lesion, so was offered an oral combination of dabrafenib and trametinib, for the recommended 12-month treatment course. He is currently doing well, recurrence-free over 5 years later, with complete regression of the intramuscular lesion, and no other lesions on annual CT scans thus far.

## Discussion and conclusions

Metastasis on presentation is a predictor of poor prognosis in MM. Generally, MM has a protracted disease course, in which patients can have a ‘disease free’ window after excision of the primary lesion, presenting with visceral metastases even decades later [9, 10]. The thickness of the primary MM lesion and the presence of a positive sentinel lymph node are the two main factors, however others have been also associated with the probability of metastatic recurrence, hence many of these are now included in the MM staging [11, 12]. Solitary lung and intramuscular metastasis in the absence of a primary MM are not at all common. Intramuscular metastases from MM are most commonly seen in previously resected tumours with high thickness, and at the site of excision. It is very rare to have an intramuscular solitary lesion without a primary MM. Solitary intramuscular metastasis from primary lung cancers are rare, with only a handful of case in the literature [13, 14], however they are usually painful and cause discomfort. In this case, the lesion was not causing pain or discomfort to the patient, and his findings were incidental during the investigations for a primary lung cancer.

The majority of pulmonary metastases are peripherally located (or even sub-pleural), which makes a wedge resection a reasonable option. Lung metastasectomy is a very attractive modality for solitary metastases in MM, as it has been associated with increased 5-year survival in such cases [15]. The mode of resection (VATS vs thoracotomy) depends on the position of the lesion, as well as the intention for lung tissue preservation in lesions <5cm, while maintaining clear and adequate resection margins, and of course the skill, experience, and preference of the surgeon. In our case, with a small (20 mm) peripheral lesion, a VATS wedge resection was performed, and a surgical margin of 9 mm was achieved, according to the histopathological report. While the literature is lacking recent meta-analyses or large series that incorporate recent advanced preoperative imaging modalities that we have available nowadays (PET, MRI etc.), when prognostic factors were evaluated, the importance of the number of pulmonary metastases was emphasised. Overwhelmingly, in nearly all previous series, patients with 1 lesion had best survival rates post-operatively. On the other hand, the type of surgical procedure (wedge resection vs lobectomy) has not been found to be important, probably highlighting that the surgical skill to ensure clear adequate margins is more important than the mode to achieve it. Usually, full lobectomies or extended anatomic resections are reserved for deeply located pulmonary metastases, or larger lesions which carry the risk of non-free margins.

To our knowledge, this is the first reported case of a MM with unknown primary, presenting as a SPN combined with solitary intramuscular metastasis (mimicking a primary lung cancer), diagnosed via US-guided core needle biopsy, and treated with lung metastasectomy and combination oral targeted therapy, with a disease-free survival of over 5 years. It certainly highlights the importance of histologic diagnosis before proceeding to surgical resection of lung lesions, despite clinical and radiologic findings supportive of a certain tumor type. Additionally, it strengthens the observation that lung metastasectomy in MM without primary can have a role in providing a decent disease-free survival, when combined with targeted oral therapy. Clinicians should remain vigilant for rare extrathoracic cancers masked as primary lung cancers in the era of widespread lung cancer screening.

## Data Availability

Not applicable.

## Abbreviations

SPN: Solitary pulmonary nodule
PET: Positron-emission tomography
CT: Computed tomography
MM: Malignant melanoma
mm: Millimeters
SUV: Standardized uptake value
HMB-45: Human melanoma black-45
TTF-1: Thyroid transcription factor 1
US: Ultrasound
